# Identifying Predictors of Unfavorable Treatment Outcomes in Tuberculosis Patients

**DOI:** 10.3390/ijerph21111454

**Published:** 2024-10-31

**Authors:** Ji Yeon Lee, Jun-Pyo Myong, Younghyun Kim, Ina Jeong, Joohae Kim, Sooim Sin, Yunhyung Kwon, Chieeun Song, Joon-Sung Joh

**Affiliations:** 1 Division of Pulmonary and Critical Care Medicine, Department of Internal Medicine, National Medical Center, Seoul 04564, Republic of Korea; fulgeo@nmc.or.kr (J.Y.L.); 1770@nmc.or.kr (Y.K.); inajeong@nmc.or.kr (I.J.); joohaekim@nmc.or.kr (J.K.); sooim216@nmc.or.kr (S.S.); 2 Department of Occupational & Environmental Medicine, Seoul St. Mary’s Hospital, College of Medicine, The Catholic University of Korea, Seoul 06591, Republic of Korea; medical001@catholic.or.kr; 3 Division of Tuberculosis Prevention and Control, Korea Disease Control and Prevention Agency, Cheongju 28159, Republic of Korea; yhhodori@korea.kr (Y.K.); cesong@korea.kr (C.S.)

**Keywords:** tuberculosis, unfavorable outcomes, multivariable logistic, regression analysis

## Abstract

Objectives: In South Korea, there has been a continuous decrease in the incidence of tuberculosis (TB) attributable to a national TB elimination program; however, TB still poses a significant socioeconomic burden. This study aimed to analyze factors associated with successful TB treatment and to identify refractory patient groups with unfavorable outcomes. Methods: We analyzed anonymized data on 89,150 patients with TB provided by the Korea Disease Control and Prevention Agency. Specifically, we collected independent variables, which were categorized as individual, regional, and medical facility factors. Individual factors included age, sex, nationality, TB type, drug-resistant status, category of TB, housing type, underlying disease status, number of referrals, and smoking status. Regional factors referred to the region where the TB case was reported. Medical facility factors included the first visit to a medical facility, categorized by hospital type and the distinction between private–public mix (PPM) and non-PPM depending on the presence or absence of dedicated TB nurses. These factors were analyzed in relation to treatment success to identify refractory patient groups with unfavorable outcomes. Results: Multivariable logistic regression analysis revealed the following significant factors associated with successful TB treatment: sex, nationality, status of drug-resistant TB, category of TB, number of referrals, region of TB registry, underlying diseases, and smoking status. Specifically, compared with their relevant counterparts, male patients had a lower rate of successful treatment (adjusted odds ratio [aOR]: 0.66, reference [Ref.]: women); Korean nationals had a higher rate of treatment success (aOR: 7.20, Ref. foreign residents in Korea); resistant TB was associated with a lower rate of treatment success (aOR: 0.35, Ref.: non-resistant TB status); newly treated patients had a higher rate of treatment success (aOR: 1.75, Ref.: retreatment patient); switching hospitals once (aOR: 1.78), never (aOR: 1.41), or twice (aOR: 1.37) was associated with increased treatment success (Ref.: three or more times); having zero (aOR: 1.45), one (aOR: 1.31), or two (aOR: 1.24) underlying diseases was associated with a higher rate of treatment success (Ref. three or more underlying diseases); and past smokers (aOR: 1.40) and non-smokers (aOR: 1.35) had a higher rate of treatment success (Ref.: current smokers). Conclusions: Our study identified several factors contributing to unfavorable treatment outcomes in tuberculosis patients, including male patients, foreign residents in Korea, drug-resistant TB, retreatment patients, frequent hospital switching, multiple underlying diseases, and current smoking status. These research findings could inform the development of efficient management strategies and policies for improving the treatment success rate among patients with TB.

## 1. Introduction

Globally, the incidence of tuberculosis (TB) has seen a cumulative decrease of 9% from 2015 to 2019. The World Health Organization (WHO) releases the Global Tuberculosis Report annually, which details the global status of TB. According to the 2022 report, the global incidence of TB was 131 per 100,000 people, with a mortality of 17.7 per 100,000 people [1]. Accordingly, TB is no longer among the top 10 causes of death worldwide and currently sits at the 13th position [1].

The incidence of tuberculosis (TB) in South Korea, as announced by the World Health Organization (WHO) for 2022, was 39 per 100,000 people, which reflects a 13.0% decrease from the previous year. However, the mortality rate from TB remained the same as the previous year at 3.8 per 100,000 people. South Korea ranks second in terms of TB incidence and fourth in TB mortality among the 38 member countries of the Organization for Economic Co-operation and Development (OECD) [2]. The Korean government has announced a strategic plan for reducing the incidence of TB to 10 per 100,000 people by 2030. To fortify its support for TB treatment, it has allocated dedicated TB nurses to various cooperating private–public mix (PPM) healthcare facilities, as well as designated specialized hospitals for managing multidrug-resistant TB and assisting economically vulnerable groups through the Tuberculosis Relief Belt Support Project [3]. The PPM project involves assigning TB management-dedicated nurses to private medical institutions to provide counseling and medication guidance and manage non-compliant TB patients. As of 2022, there are 184 medical institutions participating in the PPM, treating approximately 80% of reported TB patients in Korea. Since the official launch of the PPM project in 2011, the number of TB patients has decreased by more than 50%, from 50,419 in 2011 to 22,904 in 2021 [4]. The Tuberculosis Relief Belt Support Project is an integrated service support project for economically disadvantaged TB patients, addressing gaps in TB treatment. Since 2014, it has provided financial support for treatment costs, caregiving expenses, and transportation costs to TB patients in need. In 2022, 14 regional public medical institutions participated in the project [5].

The Korean government is making significant efforts in tuberculosis control projects, including tuberculosis prevention and early detection, patient treatment and management, and establishment and operation of an integrated tuberculosis management system [2]. Accordingly, the present study was conducted to investigate the development of management strategies for patients with TB. Specifically, this study aimed to identify factors associated with successful TB treatment and to identify refractory patient groups with unfavorable outcomes. These results could inform management measures that increase the success rates of TB treatment.

## 2. Materials and Methods

### 2.1. Participants

Data from the ‘Tuberculosis Registration Report’ and ‘Tuberculosis Case Investigation Form’, which are collected and managed by the Korea Disease Control and Prevention Agency (KDCA), were used to analyze factors associated with TB treatment outcomes. The data were confirmed patient records from the ‘Annual Report on Tuberculosis Notifications’ published yearly by the Korea Disease Control and Prevention Agency (KDCA) for the years [6,7,8] 2016–2018. From the initial anonymized dataset of 154,233 TB cases reported between 1 January 2016 and 31 December 2018, which was provided by the KDCA, we excluded 36,408 duplicate reports during the initial screening. In the second screening, we excluded 26,627 reports with missing outcomes, changed diagnoses, or deaths from causes other than TB. In this context, patients with missing final outcomes were those who were still undergoing treatment at the time the data were received, and therefore did not have a final outcome. Additionally, 2048 patients were excluded due to unclear data on sex, reporting institutions, and treatment history resulting from unperformed or erroneous case investigations. Consequently, 89,150 patients were included in the final analysis.

### 2.2. Methods

#### 2.2.1. Study Design

This study is a retrospective study conducted to identify the factors influencing TB treatment outcomes. The analysis categorized the factors into individual, regional, and medical facility factors. Among the independent variables, individual factors were age, sex, nationality, type of TB, drug resistance, category of TB, housing type, underlying diseases, number of referrals, and smoking status. Regional factors included the region of TB report, while medical facility factors included the first visit to a medical facility, categorized by hospital type and the distinction between private–public mix (PPM) and non-PPM depending on the presence or absence of dedicated TB nurses (hereinafter referred to as “PPM status”). These factors were analyzed in relation to treatment success to identify refractory patient groups with unfavorable outcomes.

#### 2.2.2. Variables

Basic information regarding the variables has already been provided above. The types of underlying diseases diagnosed before the tuberculosis diagnosis included hypertension, diabetes, dementia, Parkinson’s disease, cancers, cerebral infarction, schizophrenia, heart disease, and asthma.

Age was analyzed according to age group; sex was differentiated as male and female; nationality was categorized as locals and foreign residents; type of TB was categorized as pulmonary (PTB), extrapulmonary (EPTB), and pulmonary combined with extrapulmonary (PEPTB); and drug-resistance status was dichotomized as resistant and non-resistant. “Drug-resistant” TB included both “multidrug-resistant” and “extensively drug-resistant” TB (MDR/XDR TB), which is consistent with the KDCA annual report of the TB registry. The category of TB was categorized as “new treatment” and “retreatment”. Housing type was categorized as “with family”, “alone”, “dormitory/boarding”, and “other”. Smoking status was categorized as “Non-smoker” (never smoked), “Former smoker” (smoked in the past but quit before the onset of tuberculosis), and “Current smoker” (smoking at the time of tuberculosis diagnosis). Underlying disease status was categorized into one, two, and more than three.

Regional factors, which were based on the location of registration, included municipals and provinces such as Seoul, Busan, Daegu, Incheon, Gwangju, Daejeon, Ulsan, Sejong, Gyeonggi, Chungcheong, Jeolla, Gyeongsang, and Jeju. These were further simplified into “Seoul Capital Area”, “metropolitan cities”, and “other regions” based on the population density.

Medical facility factors were analyzed based on the first visit to the medical facilities, categorized by hospital type and PPM status. Hospital type was classified as hospital, public health center (PHC), clinic, or general hospital. PPM status was classified as PPM or non-PPM.

South Korea mandates that healthcare facilities report the treatment outcomes to the PHC in the region upon completing patient treatment [9]. In cases of drug-susceptible TB, a “cure” was indicated by a case where a patient who was confirmed to have bacterium-positive PTB at treatment initiation had a negative sputum culture test result after treatment completion (or in the last month) and who had one or more previous negative sputum culture tests. The concept of “completed treatment” applied to cases without evidence of treatment failure and where, despite the absence of a negative sputum smear and culture result after treatment completion (or in the last month), previous smear and culture tests had been negative at least once. For drug-resistant TB, “cure” was defined as cases where a patient had judiciously completed treatment and showed at least three consecutive negative results on culture tests, with a minimum 30-day interval following intensive phase treatment. The concept of “completed treatment” applied to cases where treatment was completed, but the negative standard bacterial cultures did not meet the criteria for “cure” [10]. Consistent with WHO recommendations [11], “cure” and “completed treatment” cases were combined to determine the rate of successful TB treatment in Korea. Treatment failure in drug-susceptible TB is defined as having a positive result in a sputum smear or culture performed in the fifth month or later after starting treatment. Treatment failure in drug-resistant TB occurs when treatment is terminated for one of the following reasons: failure in negative conversion by the end of the intensive treatment phase, bacteriological reversion during the maintenance phase, additional resistance acquired to fluoroquinolones or injectable drugs, side effects of TB drugs, or when a permanent change in the prescription of anti-TB drugs is required for at least two months [10].

#### 2.2.3. Data Analysis

Data regarding individual, regional, and medical facility factors are presented as percentages and frequencies, with differences according to treatment success being analyzed using the chi-square test. Multivariable logistic regression analysis was conducted to identify factors associated with treatment success. Statistical analyses were performed using the SAS statistical package program (9.4M8), with statistical significance being set at a *p*-value < 0.05. In the study, the outcome variable is the “treatment outcome”, which is encoded as follows: 0 represents “success” and 1 represents an “unfavorable outcome”.

## 3. Results

Table 1 outlines the treatment outcomes for each factor, including individual, regional, and medical facility factors. Among the 89,150 patients who were examined, the rates of treatment success and unfavorable outcomes were 94.3% and 5.7%, respectively. According to age, the treatment success rate was highest at 98.2% in the youngest age group (0−19 years) and lowest at 89.0% for individuals aged ≥ 80 years. Regarding sex, females had higher treatment success rates than males. By nationality, local residents had a higher treatment success rate than foreign residents. Regarding the TB type, EPTB had the highest treatment success rate, while regarding drug resistance, non-resistant TB had a higher treatment success rate than drug-resistant TB. Newly treated patients had a higher success rate than Retreatment patients. The number of referrals was negatively correlated with the treatment success rate. Regarding the region of the TB registry, areas outside the Capital Area had higher treatment success rates than those within the Capital Area (Seoul, Incheon, and Gyeonggi). According to housing type, patients living with family had the highest treatment success rate. The number of underlying diseases was negatively correlated with the treatment success rate; moreover, non-smokers had a higher treatment success rate than former or current smokers. Among the types of first visits to medical facilities, “clinic” showed the highest treatment success rate and “PPM” in the PPM status factor outperformed “non-PPM”.

Table 2 presents the results of the multivariable logistic regression analysis of factors affecting TB treatment success. Patients who were older, male, foreign residents, drug-resistant, retreated, with three or more referrals, living in metropolitan cities and the Seoul Capital Area (Seoul–Incheon–Gyeonggi), living in dormitories or boarding houses, with three or more underlying diseases, current smokers, and attending non-PPM hospitals were more likely to experience unfavorable treatment outcomes.

Table 3 presents the results of multivariable logistic regression analysis including cases of treatment failure and loss-to-follow-up cases and excluding patients who died. In this reanalysis, patients who were male, foreign residents, drug-resistant, retreated, with three or more referrals, living in metropolitan cities and the Seoul Capital Area (Seoul-Incheon-Gyeonggi), with three or more underlying diseases, and current smokers had a significantly lower rate of treatment success.

Table 4 presents the mitigation strategies implemented by the Korean government to improve the treatment success rate among tuberculosis patients.

## 4. Discussion

This study analyzed factors that influence treatment success in patients with TB in order to identify refractory patient groups. In South Korea, there has been a continuous decrease in the incidence of TB, which is attributable to a national TB elimination program; however, TB still poses a significant socioeconomic burden. Measures for improving treatment outcomes in patients with TB should consider both medical and social welfare; additionally, close cooperation between the two fields is required [12]. We identified several factors associated with treatment success in patients with TB, including age, sex, nationality, drug-resistant TB, category of TB, number of referrals, region of TB registry, type of housing, underlying diseases, and PPM status. Multivariable logistic regression analysis, which excluded deaths and only analyzed cases of treatment failure and loss to follow-up to assess the impact of death, identified sex, nationality, drug-resistant TB, category of TB, number of referrals, region of TB registry, underlying diseases, and smoking status as important factors. While we can control cases where patients die due to the nature of their diseases, managing patients who discontinue treatment for various reasons is challenging. Therefore, it is considered important to analyze and manage these discontinued patients.

A previous study conducted in 2014 on the factors affecting the treatment outcomes of treatment-naive patients with TB found that the treatment success rate was significantly lower with old age, male sex, unknown resident registration number, foreign nationality, culture-negative TB, extrapulmonary TB, drug-resistant TB, and one or more referrals [13]. Male sex, foreign nationality, and drug-resistant TB are consistent with the results of our study, and the management of patients with male sex, foreign nationality, and drug-resistant TB should be emphasized. In our study, regarding nationality, local residents (OR: 7.20) had a higher probability of treatment success compared with foreigners. The low treatment success rate among foreigners residing in Korea could be attributed to the fact that most foreigners receiving tuberculosis treatment are illegal residents. In a study on patients with TB among vulnerable groups in Korea (TB Safety Belt Support Project), the recipients of support for foreigners residing in Korea were illegal foreign residents. It was found that 63.5% were Koreans and 36.5% were foreigners residing in Korea [14]. This indicates that most foreigners receiving tuberculosis treatment are illegal residents, and consistent with our findings, their treatment outcomes showed a low likelihood of success. Therefore, the management of illegal foreign residents with TB is an important issue.

Regarding illegal foreign residents with TB, public health centers provide free medical treatment or support for treatment expenses through the TB Safety Belt Support Project. However, foreigners with multidrug-resistant TB who enter Korea without a visa or with a short-term visa are considered to be seeking free or subsidized treatments and are forcibly deported [9]. Accordingly, they appear to not seek treatment due to fear of deportation. Therefore, patients with multidrug-resistant TB who enter Korea without a visa or with a short-term visa should not be assumed to have entered the country for treatment. For example, if an illegal foreign resident who entered the country with no visa or a short-term visa stays in Korea for >1 year, such a stay may not be considered to be for medical treatment. Previous studies have indicated immigrants as a group at risk of multidrug-resistant TB [15]. The policy was implemented to prevent the short-term entry of multi-drug resistant tuberculosis (TB) patients into the country, but it is also considered important to devise measures to improve the treatment adherence of foreign residents with TB in the country.

Socially and economically disadvantaged groups may develop tuberculosis as a result of factors such as malnutrition, which can arise from communal living and poor living conditions. Moreover, they do not receive prompt diagnosis and treatment due to inaccessible medical care [16]. In our study, patients with drug-resistant TB had a lower probability of treatment success compared with those with non-resistant TB (aOR: 0.35). Compared with individuals aged 30–39 years, persons aged <20 years and ≥75 years had a 4.1% and 9.6% higher risk of multidrug-resistant TB, respectively, but these differences were not statistically significant. All other age groups showed a trend of reduced risk of multidrug-resistant TB with aging [17]. When mycobacteria are detected in smear tests, a drug susceptibility test (DST) is performed. Traditionally, multidrug-resistant TB has been diagnosed with a DST using a medium; however, this takes a long time to obtain results. Accordingly, genotypic DSTs are currently being used often for mycobacteria, which allow rapid verification of the results. By applying this method, DST results for isoniazid or rifampicin can be verified within 2–3 days [18]. Considering that the resistance rate to quinolones or injectable drugs among multidrug-resistant tuberculosis (MDR-TB) patients diagnosed in Korea reaches 25–33%, it is important to quickly diagnose extensively drug-resistant tuberculosis (XDR-TB). Therefore, to effectively treat MDR-TB, it is recommended to conduct rapid resistance testing for quinolone drugs and injectable drugs in patients diagnosed with rifampicin-resistant or multidrug-resistant tuberculosis [19].

Patients with multidrug-resistant TB can be classified as those who are infected with multidrug-resistant TB from the beginning (initial resistance) and those who experience resistance due to improper prescription, inappropriate drug use, and non-adherence (acquired resistance) [18]. To prevent initial resistance, it is necessary to prevent the spread of infection through prompt diagnosis and treatment of patients with multidrug-resistant TB. To prevent acquired resistance, it is necessary to ensure appropriate standards, with the prevention of single-drug treatment, irregular dosing, and inappropriate dosage [18,20]. Accordingly, it is important to closely manage patients with multidrug-resistant TB [21].

Regarding treatment classification, newly treated patients (aOR: 1.75) showed a higher probability of treatment success than previously treated patients. A short-term treatment course of 6 months is important for TB; further, 30% of patients relapse if the treatment course is not properly completed [22]. In 2020, the treatment success rate for new patients was 81.3%, while the success rate for retreatment patients was 75.2%, which is 6.1 percentage points lower than that of new patients. The treatment interruption rate was 1.8% for new patients and 3.6% for retreatment patients, which is twice as high as that of new patients. The proportion of patients undergoing treatment was 2.6% for new patients and 4.5% for retreatment patients, indicating a higher treatment interruption rate among retreatment patients [23]. The reason new patients have a higher likelihood of treatment success is thought to be due to the lower treatment adherence among retreatment patients. Most retreatment patients are those receiving treatment after previous treatment failure or interruption, and irregular medication intake is likely the primary cause.

In the analysis excluding deaths, the probability of treatment success was highest among patients with one referral (aOR: 1.78), followed by those without a referral (aOR: 1.41) and two referrals (aOR: 1.37), compared to those with three referrals. There are many reasons why patients are referred. Patients referred for treatment can be categorized based on the nature of their diseases and service-related reasons. Patients referred due to the nature of their diseases are often transferred because their original hospital lacks the necessary medical resources, necessitating treatment at a higher-level hospital. In such cases, the likelihood of treatment disruption is low since the patients are sent to facilities equipped with the resources they need. In contrast, patients referred for service-related reasons might seek transfer to hospitals where they can receive financial support, especially in cases of economic hardship. Additionally, non-compliant patients who engage in behaviors such as drinking, smoking, or assaulting medical staff may face treatment refusal and subsequent referral. Economically disadvantaged patients may abandon treatment due to financial burdens, while non-compliant patients might not establish rapport with medical staff, increasing the risk of treatment dropout. Tuberculosis mostly occurs in the lower socio-economic classes, which is related to socioeconomic vulnerabilities such as malnutrition due to poverty, smoking, high-risk drinking, and overcrowding. Additionally, a lack of social networks, social exclusion, limited access to healthcare, and a lack of trust and understanding of institutions due to vulnerability are factors that hinder tuberculosis treatment for vulnerable groups [24]. Thus, active management of patients referred for service-related reasons is crucial.

The probability of treatment success was highest among individuals without underlying diseases (aOR: 1.45), followed by those with one underlying disease (aOR: 1.31) and two underlying diseases (aOR: 1.24), compared with those with three or more underlying diseases. Notably, our findings showed that patients with three or more underlying diseases had a lower chance of treatment success in the multivariable regression analysis excluding death cases. This also means that patients with underlying diseases have a higher likelihood of treatment failure or discontinuation.

Regarding the use of medical care, basic life beneficiaries, those with single-person households, smokers, individuals with poor subjective health, and males are less likely to visit medical institutions when they have a medical condition [25]. Patients with three or more underlying diseases often have poor subjective health outcomes. Government subsidies have made TB treatment less burdensome for poor groups. However, since the government does not subsidize treatment for underlying diseases unrelated to TB, low-income patients may not receive proper treatment due to the burden of treatment costs, which may reduce the chances of treatment success [14].

In our study, former smokers (aOR: 1.40) and non-smokers (aOR: 1.35) were more likely to have treatment success compared with current smokers. A cross-sectional study on urban communities in South Africa showed a two-fold increase in TB infections in smokers than in non-smokers [26]. Among types of TB, EPTB had a higher treatment success rate than PTB, but this difference was not statistically significant. This may be because the mortality rate for pulmonary tuberculosis patients is 2.7%, whereas it is 1.0% for extrapulmonary tuberculosis patients. Tuberculosis is classified into PTB and EPTB based on the location of the lesions. Extrapulmonary tuberculosis occurs when the disease affects organs other than the lung parenchyma, such as the pleura, lymph nodes, abdominal cavity, or skeletal muscles [2].

Diagnosing extrapulmonary tuberculosis is relatively challenging compared to pulmonary tuberculosis. Pulmonary tuberculosis can often be easily diagnosed with a chest X-ray and sputum tests (smear and culture) but diagnosing extrapulmonary tuberculosis often requires invasive procedures to obtain specimens. The culture positivity rate for tests diagnosing extrapulmonary tuberculosis is about 40–70%, which is lower than for pulmonary tuberculosis. However, in tissue biopsies, chronic granulomatous inflammation with necrosis is observed in 70–80% of cases, making histological findings useful for the rapid diagnosis of extrapulmonary tuberculosis [27].

The incidence of extrapulmonary tuberculosis has remained at a consistent proportion among all tuberculosis patients. Treatment for extrapulmonary tuberculosis is similar to that for pulmonary tuberculosis, but in some cases (e.g., tuberculous meningitis and tuberculous pericarditis), adjunctive use of steroids may be beneficial. Unlike pulmonary tuberculosis, extrapulmonary tuberculosis shows a higher frequency of paradoxical reactions due to immunological mechanisms, which can make it challenging to distinguish between treatment failure and relapse during or after treatment. Therefore, for patients suspected of having extrapulmonary tuberculosis, obtaining possible specimens for microbiological diagnosis and conducting drug susceptibility tests are helpful in resolving these clinical challenges [28]. Compared with other regions, metropolitan cities had the lowest treatment success likelihood (OR: 0.76), followed by the Seoul Capital Area (OR: 0.63), which could be attributed to the higher treatment discontinuation rates in the Seoul Capital Area. The high discontinuation rate in the metropolitan area is thought to be influenced by housing types due to the population density in this region. In Table 1 of this study, it is shown that the discontinuation rate for those living in dormitory/boarding situations is 12.4%. Patients who discontinue TB treatment can infect others if they ignore government guidelines by using public transport or visiting crowded areas while carrying mycobacteria. Therefore, further studies on patients who discontinue TB treatment are warranted.

This study has several limitations. First, we did not present the results by separately classifying new and Retreatment patients. Second, smoking history was determined from case reports of patients with TB prepared prior to treatment initiation. Therefore, it is difficult to determine the causal relationship with the treatment outcomes. Third, although the socioeconomic status of patients with TB influences treatment outcomes, we did not identify the regional deprivation index. Fourth, mortality among patients with TB is categorized as TB-related or non-TB-related; however, we only analyzed the outcomes for TB-related deaths. Fifth, we classified drug-resistant TB by including only multidrug-resistant and extensively drug-resistant TB, and we did not consider isoniazid-resistant TB.

## 5. Conclusions

Analyzing and managing patients with a low probability of treatment success is an important strategy for eliminating TB. These findings can provide valuable information for management measures aimed at increasing TB treatment success rates.

According to our research, foreign patients residing in Korea, those with drug-resistant TB, those who have received three or more referrals, those living in Seoul, Gyeonggi, or metropolitan areas, those with three or more underlying diseases, and current smokers had relatively low probabilities of treatment success.

It is necessary to develop measures to improve treatment adherence among foreign TB patients residing in Korea. There should be a policy to support the cost of rapid drug susceptibility testing for all TB patients to diagnose multi-drug-resistant TB. Additionally, TB screening should be strengthened for groups living in dormitory-style accommodations in metropolitan areas. Intensive management is required for patients who are transferred for service-related reasons and those with underlying diseases. Lastly, further research on patients who discontinue treatment is needed to manage non-adherent patients.

## Figures and Tables

**Table 1:** Factor-specific distribution of the patients with TB (2016−2018).

Variable	Category	Total	Treatment Success			Unfavorable Outcome				*p*
			Sum	Cure	Complete	Sum	Failure	Lost to Follow-Up	Death	
Age	0−19	2130 (100.0)	2090 (98.2)	457 (21.5)	1633 (76.7)	40 (1.8)	0 (0)	37 (1.7)	3 (0.1)	<0.0001
	20−29	8382 (100.0)	7935 (94.7)	1844 (22.0)	6091 (72.7)	447 (5.3)	3 (0.0)	433 (5.2)	11 (0.1)	
	30−39	8888 (100.0)	8471 (95.3)	1973 (22.2)	6498 (73.1)	417 (4.7)	4 (0.0)	381 (4.3)	32 (0.4)	
	40−49	12,094 (100.0)	11,519 (95.3)	2597 (21.5)	8922 (73.8)	575 (4.7)	6 (0.0)	466 (3.9)	103 (0.8)	
	50−59	16,390 (100.0)	15,554 (94.9)	3445 (21.0)	12,109 (73.9)	836 (5.1)	9 (0.1)	610 (3.7)	217 (1.3)	
	60−69	14,071 (100.0)	13,414 (95.3)	2906 (20.6)	10,508 (74.7)	657 (4.7)	3 (0.0)	415 (3.0)	239 (1.7)	
	70−79	15,476 (100.0)	14,681 (94.9)	3256 (21.0)	11,425 (73.9)	795 (5.1)	4 (0.0)	296 (1.9)	495 (3.2)	
	80+	11,719 (100.0)	10,427 (89.0)	2059 (17.6)	8368 (71.4)	1292 (11.0)	1 (0.0)	237 (2.0)	1054 (9.0)	
Sex	Male	52,830 (100.0)	49,453 (93.6)	11,725 (22.2)	37,728 (71.4)	3377 (6.4)	27 (0.0)	1989 (3.8)	1361 (2.6)	<0.0001
	Female	36,320 (100.0)	34,638 (95.4)	6812 (18.8)	27,826 (76.6)	1682 (4.6)	3 (0.0)	886 (2.4)	793 (2.2)	
Nationality	Locals	83,546 (100.0)	79,621 (95.3)	17,217 (20.6)	62,404 (74.7)	3925 (4.7)	25 (0.0)	1771 (2.1)	2129 (2.6)	<0.0001
	For. Res.	5604 (100.0)	4470 (79.8)	1320 (23.6)	3150 (56.2)	1134 (20.2)	5 (0.1)	1104 (19.7)	25 (0.4)	
Type of TB	PTB	66,376 (100.0)	62,389 (94.0)	16,481 (24.8)	45,905 (69.2)	3990 (6.0)	30 (0.1)	2150 (3.2)	1810 (2.7)	<0.0001
	EPTB	18,057 (100.0)	17,266 (95.6)	1209 (6.7)	16,057 (88.9)	791 (4.4)	0 (0.0)	609 (3.4)	182 (1.0)	
	PEPTB	4717 (100.0)	4439 (94.1)	847 (18.0)	3592 (76.1)	278 (5.9)	0 (0.0)	116 (2.5)	162 (3.4)	
Drug-resistance	Resistant	2079 (100.0)	1722 (82.8)	692 (33.3)	1030 (49.5)	357 (17.2)	14 (0.7)	248 (11.9)	95 (4.6)	<0.0001
	Non-res.	87,071 (100.0)	82,369 (94.6)	17,845 (20.5)	64,524 (74.1)	4702 (5.4)	16 (0.0)	2627 (3.0)	2059 (2.4)	
Category of TB	New Tx	76,570 (100.0)	72,647 (94.9)	15,852 (20.7)	56,795 (74.2)	3923 (5.1)	18 (0.0)	2111 (2.8)	1794 (2.3)	<0.0001
	Re-Tx	12,580 (100.0)	11,444 (91.0)	2685 (21.4)	8759 (69.6)	1136 (9.0)	12 (0.1)	764 (6.0)	360 (2.9)	
Number of referrals	0	68,228 (100.0)	64,561 (94.6)	13,719 (20.1)	50,842 (74.5)	3667 (5.4)	15 (0.0)	2107 (3.1)	1545 (2.3)	<0.0001
	1	16,104 (100.0)	15,090 (93.7)	3707 (23.0)	11,383 (70.7)	1014 (6.3)	6 (0.0)	539 (3.4)	469 (2.9)	
	2	3609 (100.0)	3353 (92.9)	828 (22.9)	2525 (70.0)	256 (7.1)	3 (0.1)	160 (4.4)	93 (2.6)	
	≥3	1209 (100.0)	1087 (89.9)	283 (23.4)	804 (66.5)	122 (10.1)	6 (0.5)	69 (5.7)	47 (3.9)	
Registered region	Seoul	22,342 (100.0)	20,955 (93.8)	4927 (22.1)	16,028 (71.7)	1387 (6.2)	12 (0.1)	949 (4.2)	426 (1.9)	<0.0001
	Busan	5975 (100.0)	5694 (95.3)	1305 (21.8)	4389 (73.5)	281 (4.7)	1 (0.0)	135 (2.3)	145 (2.4)	
	Daegu	6510 (100.0)	6116 (94.0)	437 (6.7)	5679 (87.3)	394 (6.0)	0 (0.0)	229 (3.5)	165 (2.5)	
	Incheon	4555 (100.0)	4259 (93.5)	792 (17.4)	3467 (76.1)	296 (6.5)	0 (0.0)	144 (3.2)	152 (3.3)	
	Gwangju	2988 (100.0)	2858 (95.7)	940 (31.5)	1918 (64.2)	130 (4.3)	0 (0.0)	49 (1.6)	81 (2.7)	
	Daejeon	2959 (100.0)	2844 (96.1)	399 (13.5)	2445 (82.6)	115 (3.9)	0 (0.0)	51 (1.7)	64 (2.2)	
	Ulsan	1953 (100.0)	1863 (95.4)	541 (27.7)	1322 (67.7)	90 (4.6)	0 (0.0)	50 (2.6)	40 (2.0)	
	Sejong	43 (100.0)	39 (90.6)	13 (30.2)	26 (60.4)	4 (9.4)	0 (0.0)	2 (4.7)	2 (4.7)	
	Gyeonggi	17,772 (100.0)	16,652 (93.7)	3532 (19.9)	13,120 (73.8)	1120 (6.3)	8 (0.0)	706 (4.0)	406 (2.3)	
	Gangwon	3459 (100.0)	3251 (94.0)	1346 (38.9)	1905 (55.1)	208 (6.0)	1 (0.0)	81 (2.3)	126 (3.7)	
	Ch’cheong	5023 (100.0)	4751 (94.6)	1224 (24.4)	3526 (70.2)	273 (5.4)	1 (0.0)	112 (2.2)	160 (3.2)	
	Jeolla	5813 (100.0)	5531 (95.1)	1209 (20.8)	4322 (74.3)	282 (4.9)	4 (0.1)	129 (2.2)	149 (2.6)	
	Gy’sang	8708 (100.0)	8268 (94.9)	1519 (17.4)	6749 (77.5)	440 (5.1)	2 (0.0)	208 (2.4)	230 (2.7)	
	Jeju	1045 (100.0)	1006 (96.3)	352 (33.7)	654 (62.6)	39 (3.7)	1 (0.0)	30 (2.9)	8 (0.8)	
Housing type	With family	41,696 (100.0)	40,208 (96.4)	8816 (21.1)	31,392 (75.3)	1488 (3.6)	9 (0.0)	803 (2.0)	676 (1.6)	<0.0001
	Alone	43,533 (100.0)	40,478 (93.0)	9080 (20.9)	31,398 (72.1)	3055 (7.0)	21 (0.0)	1859 (4.3)	1175 (2.7)	
	Dorm	667 (100.0)	581 (87.1)	130 (19.5)	451 (67.6)	86 (12.9)	0 (0.0)	83 (12.4)	3 (0.5)	
	Other	3254 (100.0)	2824 (86.8)	511 (15.7)	2313 (71.1)	430 (13.2)	0 (0.0)	130 (4.0)	300 (9.2)	
Underlying diseases	0	50,845 (100.0)	48,150 (94.7)	11,171 (22.0)	36,979 (72.7)	2695 (5.3)	20 (0.0)	1902 (3.8)	773 (1.5)	<0.0001
	1	22,877 (100.0)	21,551 (94.2)	4641 (20.3)	16,910 (73.9)	1326 (5.8)	7 (0.0)	597 (2.6)	722 (3.2)	
	2	10,037 (100.0)	9424 (93.9)	1843 (18.4)	7581 (75.5)	613 (6.1)	3 (0.0)	233 (2.3)	377 (3.8)	
	≥3	5391 (100.0)	4966 (92.1)	882 (16.3)	4084 (75.8)	425 (7.9)	0 (0.0)	143 (2.7)	282 (5.2)	
Smoking status	Non-sm.	55,887 (100.0)	52,911 (94.7)	10,847 (19.4)	42,064 (75.3)	2976 (5.3)	8 (0.0)	1564 (2.8)	1404 (2.5)	<0.0001
	Former.	15,493 (100.0)	14,637 (94.5)	3465 (22.4)	11,172 (72.1)	856 (5.5)	7 (0.0)	435 (2.8)	414 (2.7)	
	Current	17,237 (100.0)	16,121 (93.5)	4114 (23.9)	12,007 (69.6)	1116 (6.5)	15 (0.1)	831 (4.8)	270 (1.6)	
FVF (by hospital type)	Hospital	7643 (100.0)	7040 (92.1)	1817 (23.8)	5223 (68.3)	603 (7.9)	8 (0.1)	314 (4.1)	281 (3.7)	<0.0001
	PHC	6513 (100)	5996 (92.1)	2793 (42.9)	3203 (49.2)	517 (7.9)	4 (0.0)	490 (7.5)	23 (0.4)	
	Clinic	3320 (100.0)	3165 (95.3)	977 (29.4)	2188 (65.9)	155 (4.7)	0 (0.0)	144 (4.4)	11 (0.3)	
	General hospital	71,674 (100.0)	67,890 (94.7)	12,950 (18.1)	54,940 (76.6)	3784 (5.3)	18 (0.0)	1927 (2.7)	1839 (2.6)	
FVF (PPM status)	PPM	58,482 (100.0)	55,494 (94.9)	10,604 (18.1)	44,890 (76.8)	2988 (5.1)	18 (0.0)	1585 (2.7)	1385 (2.4)	<0.0001
	non -PPM	30,668 (100.0)	28,597 (93.3)	7933 (25.9)	20,664 (67.4)	2071 (6.7)	12 (0.0)	1290 (4.2)	769 (2.5)	
Total		89,150 (100.0)	84,091 (94.3)	18,537 (20.8)	65,554 (73.5)	5059 (5.7)	30 (0.0)	2875 (3.3)	2154 (2.4)	

Statistical analysis was conducted using the chi-square test (*p* < 0.05). Abbreviations: FVF, first-visit medical facilities; EPTB, extrapulmonary TB; PTB, pulmonary tuberculosis; PEPTB, pulmonary combined with extrapulmonary tuberculosis; For. Res, foreign resident; Tx, treatment; PPM, private–public mix.

**Table 2:** Results of multivariable logistic regression analysis of factors related to treatment success. Dependent variable: success (cure or completion), unfavorable outcome (failure, loss to follow-up, and death).

Category		aOR	95% CI
Age	0−19	6.95	(4.98−9.70)
	20−29	4.52	(3.94−5.17)
	30−39	4.44	(3.88−5.09)
	40−49	3.72	(3.31−4.18)
	50−59	3.63	(3.27−4.03)
	60−69	3.43	(3.08−3.81)
	70−79	2.41	(2.19−2.65)
	≥80	1.00	
sex	Male	0.66	(0.61−0.71)
	Female	1.00	
Nationality	Locals	7.20	(6.59−7.88)
	For. Res.	1.00	
Type of TB	PTB	1.11	(0.97−1.27)
	EPTB	1.28	(1.10−1.48)
	PEPTB	1.00	
Drug-resistance	resistant TB	0.35	(0.30−0.40)
	non-resistant TB	1.00	
Category of TB	New treatment	1.75	(1.62−1.89)
	Retreatment	1.00	
Number of referrals	None	1.35	(1.10−1.66)
	1	1.50	(1.21−1.84)
	2	1.42	(1.12−1.81)
	≥3	1.00	
Registered region	Seoul Capital Area	0.73	(0.67−0.78)
	Metropolitan cities	0.84	(0.77−0.91)
	Other regions	1.00	
Housing type	With family	3.16	(2.78−3.59)
	Alone	1.79	(1.59−2.02)
	Dorm	1.59	(1.21−2.10)
	Other	1.00	
Underlying diseases	0	1.45	(1.29−1.64)
	1	1.28	(1.14−1.44)
	2	1.26	(1.10−1.43)
	≥3	1.00	
Smoking status	Non-smoker	1.20	(1.10−1.30)
	Former smoker	1.33	(1.21−1.47)
	Current smoker	1.00	
FVF (by hospital type)	Hospital	0.80	(0.72−0.89)
	PHC	1.06	(0.94−1.20)
	Clinic	1.48	(1.22−1.80)
	General hospital	1.00	
FVF (PPM status)	PPM	1.12	(1.04−1.21)
	non-PPM	1.00	

Abbreviations: FVF, first-visit medical facilities; PPM, private–public mix; PHC, public health center; PTB, pulmonary tuberculosis; ETB, extrapulmonary TB; PEPTB, pulmonary combined with extrapulmonary TB.

**Table 3:** Results of the multivariable logistic regression analysis of factors related to treatment success. Dependent variable: Success (cure, completion), Unfavorable outcome (failure, loss to follow-up).

Category		aOR	95% CI
Age	0−19	1.52	(1.05−2.19)
	20−29	1.13	(0.93−1.36)
	30−39	1.15	(0.95−1.39)
	40−49	1.03	(0.86−1.23)
	50−59	1.09	(0.92−1.29)
	60−69	1.15	(0.97−1.36)
	70−79	1.24	(1.04−1.48)
	≥80	1.00	
Sex	Male	0.79	(0.71−0.87)
	Female	1.00	
Nationality	Locals	9.76	(8.85−10.77)
	For. Res.	1.00	
Type of TB	PTB	1.01	(0.83−1.23)
	EPTB	0.68	(0.56−0.84)
	PEPTB	1.00	
Drug-resistance	Resistant TB	0.38	(0.32−0.44)
	Non-resistant TB	1.00	
Category of TB	New treatment	2.30	(2.09−2.53)
	Retreatment	1.00	
Number of referrals	None	1.41	(1.08−1.85)
	1	1.78	(1.35−2.35)
	2	1.37	(1.00−1.88)
	≥3	1.00	
Registered region	Seoul Capital Area	0.63	(0.57−0.70)
	Metropolitan cities	0.76	(0.67−0.86)
	Other regions	1.00	
Housing type	With family	1.91	(1.53−2.39)
	Alone	0.98	(0.79−1.22)
	Dorm	0.92	(0.66−1.28)
	Other	1.00	
Underlying diseases	0	1.45	(1.20−1.75)
	1	1.31	(1.08−1.58)
	2	1.24	(1.00−1.54)
	≥3	1.00	
Smoking status	Non-smoker	1.35	(1.21−1.50)
	Former smoker	1.40	(1.23−1.58)
	Current smoker	1.00	
FVF (by hospital type)	Hospital	0.68	(0.58−0.78)
	PHC	0.65	(0.56−0.75)
	Clinic	0.94	(0.76−1.17)
	General hospital	1.00	
FVF (PPM status)	PPM	1.04	(0.93−1.16)
	non-PPM	1.00	

Abbreviations: FVF, first-visit medical facilities; TB, tuberculosis; EPTB, extrapulmonary TB, PEPTB, pulmonary combined with extrapulmonary TB; PPM, private–public mix; PHC, primary healthcare.

**Table 4:** National Tuberculosis Control Project (Korea).

Classification	Definition Contents
Tuberculosis prevention and early detection	On-site tuberculosis screening project, Latent tuberculosis infection management, Epidemiological investigation of tuberculosis, Tuberculosis management for foreigners residing in Korea
Patient Treatment and Management	PPM project operation, Multi-drug resistance consortium operation, Tuberculosis Safety Belt Project operation, Tuberculosis patient facility operation support
Integrated TB Management System Development and Operation	Tuberculosis integrated management system, Tuberculosis ZERO website, Tuberculosis ZERO mobile application (medication management) management
Essential TB Resources Supply	Management of Latent TB Infection Diagnostic Supplies (PPD) Supply and Management of TB Medications
Tuberculosis Awareness and Promotion	World TB Day (24 March) and TB Prevention Week Events Promotion for TB Eradication

Source: Korea Disease Control and Prevention Agency. National tuberculosis control guidelines 2024.

## Data Availability

The data are available from KCDA, but restrictions apply to the availability of these data, which were used under license for the current study, and therefore are not publicly available. However, data are available from the corresponding author upon reasonable request and with permission from KCDA.
